# *S*,*S*′-(Pyridazine-3,6-di­yl)dithio­uronium dichloride methanol monosolvate

**DOI:** 10.1107/S1600536811018514

**Published:** 2011-05-20

**Authors:** Jörg Hübscher, Lidiya Izotova, Samat Talipov, Felix Katzsch, Edwin Weber

**Affiliations:** a Institut für Organische Chemie, TU Bergakademie Freiberg, Leipziger Strasse 29, D-09596 Freiberg/Sachsen, Germany; b Institute of Bioorganic Chemistry, Academy of Sciences of Uzbekistan, M. Ulugbek Str. 83, 100125 Tashkent, Uzbekistan

## Abstract

In the title compound, C_6_H_10_N_6_S_2_^2+^·2Cl^−^·CH_3_OH, the pyrid­azine ring is almost planar, the greatest deviation from the mean plane being 0.025 (2) Å for one of the ring N atoms. The two thiouronium substituents are tilted out of this plane by 60.87 (6) and 57.94 (7)°. The thiouronium cations and the chloride anions are linked by strong N—H⋯Cl hydrogen bonds. The methanol solvent mol­ecule inter­acts with both the chloride ion (through an O—H⋯Cl hydrogen bond) and the cation (through an N—H⋯O hydrogen bond), resulting in a three-dimensional supra­molecular arrangement.

## Related literature

For pharmacological applications of pyridazine derivatives, see: Cignarella & Barlocco (2002 ▶). For details of the preparation, see: Kumagai (1960 ▶); Steck & Brundage (1959 ▶).
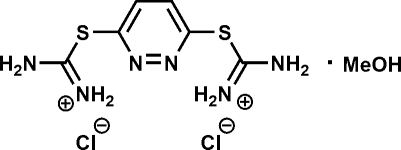


## Experimental

### 

#### Crystal data

C_6_H_10_N_6_S_2_^2+^·2Cl^−^·CH_4_O
*M* *_r_* = 333.26
Triclinic, 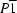

*a* = 6.7457 (2) Å
*b* = 9.0234 (3) Å
*c* = 13.0165 (4) Å
α = 104.148 (2)°
β = 98.066 (2)°
γ = 108.695 (2)°
*V* = 706.81 (4) Å^3^
*Z* = 2
Mo *K*α radiation
μ = 0.75 mm^−1^
*T* = 293 K
0.45 × 0.10 × 0.05 mm

#### Data collection

Stoe IPDS 2 diffractometer
Absorption correction: integration (*X-RED*; Stoe & Cie, 2002 ▶) *T*_min_ = 0.739, *T*_max_ = 0.959
3234 measured reflections
3234 independent reflections
2765 reflections with *I* > 2σ(*I*)
*R*_int_ = 0.071

#### Refinement

*R*[*F* ^2^ > 2σ(*F* ^2^)] = 0.030
*wR*(*F* ^2^) = 0.075
*S* = 1.03
3234 reflections
201 parameters
H atoms treated by a mixture of independent and constrained refinement
Δρ_max_ = 0.27 e Å^−3^
Δρ_min_ = −0.33 e Å^−3^

### 

Data collection: *X-AREA* (Stoe &Cie, 2002 ▶); cell refinement: *X-AREA*; data reduction: *X-RED32* (Stoe & Cie, 2002 ▶); program(s) used to solve structure: *SHELXS97* (Sheldrick, 2008 ▶); program(s) used to refine structure: *SHELXL97* (Sheldrick, 2008 ▶); molecular graphics: *XP* (Siemens, 1994 ▶); software used to prepare material for publication: *SHELXTL* (Sheldrick, 2008 ▶).

## Supplementary Material

Crystal structure: contains datablocks I, global. DOI: 10.1107/S1600536811018514/fj2416sup1.cif

Structure factors: contains datablocks I. DOI: 10.1107/S1600536811018514/fj2416Isup2.hkl

Additional supplementary materials:  crystallographic information; 3D view; checkCIF report

## Figures and Tables

**Table 1:** Hydrogen-bond geometry (Å, °)

*D*—H⋯*A*	*D*—H	H⋯*A*	*D*⋯*A*	*D*—H⋯*A*
O1*G*—H1*G*⋯Cl1^i^	0.89 (3)	2.22 (3)	3.1038 (15)	171 (3)
N3—H3*A*⋯Cl2	0.92 (3)	2.28 (3)	3.1746 (17)	166 (2)
N3—H3*B*⋯O1*G*	0.89 (3)	1.95 (3)	2.839 (2)	171 (3)
N4—H4*A*⋯Cl1	0.86 (3)	2.70 (3)	3.3950 (16)	139 (2)
N4—H4*B*⋯Cl1^i^	0.91 (3)	2.36 (3)	3.2522 (15)	167 (2)
N5—H5*A*⋯Cl1	0.89 (2)	2.39 (2)	3.2614 (16)	170 (2)
N5—H5*B*⋯Cl2^ii^	0.90 (3)	2.25 (3)	3.1413 (17)	173 (2)
N6—H6*A*⋯Cl2^iii^	0.83 (3)	2.36 (3)	3.1878 (19)	175 (3)
N6—H6*B*⋯O1*G*^iv^	0.90 (3)	2.17 (2)	2.891 (2)	136 (2)

Symmetry codes: (i) 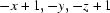; (ii) 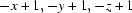; (iii) 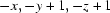; (iv) 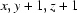.

**Fig. 1.**
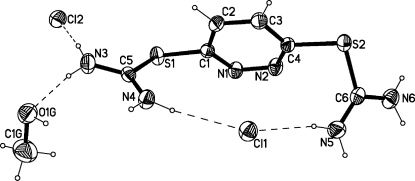
Perspective view of the title compound, showing 50% probability displacement ellipsoids for the non-H atoms. Dashed lines represent hydrogen bond.

**Fig. 2.**
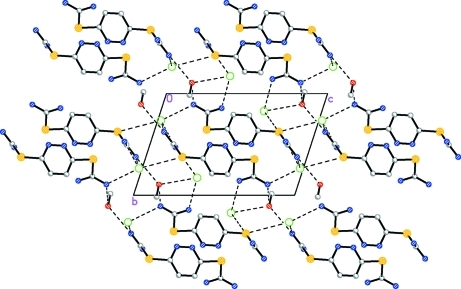
Packing diagram of the title compound viewed down the a axis. The weak hydrogen bonds are shown as dashed lines. H-atoms are omited for clarity

## supplementary crystallographic information

### Comment

Pyridazine derivatives are important compounds for pharmacological applications, among them 3,6-dithiopyridazine being a secondary substance of the title compound (Cignarella & Barlocco, 2002).

In the title compound (Fig. 1), the pyridazine ring is almost planar, with maximal deviation from the mean plane 0.025 (2) Å. Dihedral angles between the pyridazine plane and thiourea substitutes are different (S2 C6 N5 N6 - 60.87 (6)° and S1 C5 N3 N4 - 57.94 (7)°).

The structure is formed by a (C_6_H_10_N_6_S_2_)^2+^ cation and two Cl^-^ anions, connected through strong N—H···Cl^-^ hydrogen bonds [H3A···Cl2 = 2.28 (3) Å, N3—H3A···Cl2 = 166 (2)°; H4A···Cll = 2.70 (3) Å, N4—H4A···Cl1 = 139 (2)°; H5A···Cl1 = 2.39 (3) Å, N5—H5A···Cl1 = 170 (2)°], and one solvate CH_3_OH molecule bonded to the anion through a hydrogen bond [H3B···O1G = 2.839 (2) Å, N3—H3B···O1G = 171 (3)°]. Intermolecular hydrogen bonds link the Cl^-^ anions and the solvate MeOH molecule to two additional cations (Table 1) resulting in a three-dimensional supramolecular arrangement (Fig.2).

### Experimental

The title compound has been obtained as an intermediate substance of the synthesis of 3,6-dithiopyridazine involving the reaction of 3,6-dichloropyridazine with thiourea in methanol solution and was isolated before alkaline treatment (Steck & Brundage, 1959); (Kumagai, 1960). The colourless plate-type single crystals are stable in the air.

### Refinement

All H-atoms, except H-atoms involved in H-bonding, were positioned geometrically and allowed to ride on their parent atoms, with C—H=0.95 Å and *U*_iso_ = 1.2–1.5 *U*_eq_ (parent atom). The rest H-atoms were located in a difference map and fully refined

### Crystal data

**Table:**

C_6_H_10_N_6_S_2_^2+^·2Cl^−^·CH_4_O	*Z* = 2
*M**_r_* = 333.26	*F*(000) = 344
Triclinic, *P*1	*D*_x_ = 1.566 Mg m^−^^3^
Hall symbol: -P 1	Mo *K*α radiation, λ = 0.71073 Å
*a* = 6.7457 (2) Å	Cell parameters from 32534 reflections
*b* = 9.0234 (3) Å	θ = 1.7–29.6°
*c* = 13.0165 (4) Å	µ = 0.75 mm^−^^1^
α = 104.148 (2)°	*T* = 293 K
β = 98.066 (2)°	Plate, colourless
γ = 108.695 (2)°	0.45 × 0.10 × 0.05 mm
*V* = 706.81 (4) Å^3^	

### Data collection

**Table:**

Stoe IPDS 2 diffractometer	3234 independent reflections
Radiation source: sealed X-ray tube, long-fine focus	2765 reflections with *I* > 2σ(*I*)
plane graphite	*R*_int_ = 0.071
Detector resolution: 6.67 pixels mm^-1^	θ_max_ = 27.5°, θ_min_ = 2.5°
rotation method scans	*h* = −8→8
Absorption correction: integration (*X-RED*; Stoe & Cie, 2002)	*k* = −11→11
*T*_min_ = 0.739, *T*_max_ = 0.959	*l* = 0→16
3234 measured reflections	

### Refinement

**Table:**

Refinement on *F*^2^	Secondary atom site location: difference Fourier map
Least-squares matrix: full	Hydrogen site location: inferred from neighbouring sites
*R*[*F*^2^ > 2σ(*F*^2^)] = 0.030	H atoms treated by a mixture of independent and constrained refinement
*wR*(*F*^2^) = 0.075	*w* = 1/[σ^2^(*F*_o_^2^) + (0.0481*P*)^2^ + 0.1092*P*] where *P* = (*F*_o_^2^ + 2*F*_c_^2^)/3
*S* = 1.03	(Δ/σ)_max_ = 0.001
3234 reflections	Δρ_max_ = 0.27 e Å^−^^3^
201 parameters	Δρ_min_ = −0.33 e Å^−^^3^
0 restraints	Extinction correction: *SHELXL97* (Sheldrick, 2008), Fc^*^=kFc[1+0.001xFc^2^λ^3^/sin(2θ)]^-1/4^
Primary atom site location: structure-invariant direct methods	Extinction coefficient: 0.055 (4)

### Special details

**Table:**

Geometry. All s.u.'s (except the s.u. in the dihedral angle between two l.s. planes) are estimated using the full covariance matrix. The cell s.u.'s are taken into account individually in the estimation of s.u.'s in distances, angles and torsion angles; correlations between s.u.'s in cell parameters are only used when they are defined by crystal symmetry. An approximate (isotropic) treatment of cell s.u.'s is used for estimating s.u.'s involving l.s. planes.
Refinement. Refinement of *F*^2^ against ALL reflections. The weighted *R*-factor *wR* and goodness of fit *S* are based on *F*^2^, conventional *R*-factors *R* are based on *F*, with *F* set to zero for negative *F*^2^. The threshold expression of *F*^2^ > 2σ(*F*^2^) is used only for calculating *R*-factors(gt) *etc*. and is not relevant to the choice of reflections for refinement. *R*-factors based on *F*^2^ are statistically about twice as large as those based on *F*, and *R*- factors based on ALL data will be even larger.

### Fractional atomic coordinates and isotropic or equivalent isotropic displacement parameters (Å^2^)

**Table:**

	*x*	*y*	*z*	*U*_iso_*/*U*_eq_	
Cl1	0.46287 (7)	0.17255 (5)	0.64021 (3)	0.03256 (12)	
Cl2	0.10144 (7)	0.26455 (6)	0.02016 (4)	0.03622 (12)	
O1G	0.5002 (2)	−0.10500 (17)	0.13659 (11)	0.0364 (3)	
C1G	0.7040 (4)	0.0147 (3)	0.1460 (2)	0.0570 (6)	
H1G1	0.7251	0.1146	0.2013	0.085*	
H1G2	0.7098	0.0358	0.0775	0.085*	
H1G3	0.8152	−0.0251	0.1658	0.085*	
H1G	0.518 (5)	−0.132 (4)	0.197 (2)	0.059 (8)*	
H4B	0.434 (4)	0.053 (3)	0.357 (2)	0.048 (7)*	
H5A	0.436 (4)	0.388 (3)	0.780 (2)	0.037 (6)*	
H6B	0.410 (4)	0.684 (3)	0.992 (2)	0.040 (6)*	
H3A	0.201 (4)	0.137 (3)	0.138 (2)	0.046 (6)*	
H4A	0.441 (5)	0.205 (4)	0.439 (2)	0.059 (8)*	
H5B	0.581 (4)	0.541 (3)	0.880 (2)	0.047 (7)*	
H3B	0.330 (4)	0.032 (3)	0.169 (2)	0.056 (8)*	
H6A	0.183 (5)	0.646 (3)	0.947 (2)	0.049 (7)*	
S1	0.22215 (7)	0.35351 (5)	0.32279 (3)	0.02800 (11)	
S2	0.02098 (7)	0.37603 (6)	0.77378 (3)	0.03286 (12)	
C4	0.0693 (2)	0.36263 (19)	0.64195 (12)	0.0231 (3)	
N1	0.2518 (2)	0.48701 (16)	0.53011 (11)	0.0265 (3)	
N2	0.2047 (2)	0.49603 (17)	0.62795 (11)	0.0284 (3)	
C3	−0.0424 (3)	0.2143 (2)	0.55821 (13)	0.0277 (3)	
H3	−0.1409	0.1249	0.5703	0.033*	
C1	0.1553 (2)	0.34686 (18)	0.44932 (12)	0.0220 (3)	
N6	0.2904 (3)	0.6233 (2)	0.93770 (13)	0.0357 (3)	
C6	0.2824 (3)	0.50356 (19)	0.85521 (13)	0.0261 (3)	
C2	−0.0002 (3)	0.2071 (2)	0.45784 (13)	0.0271 (3)	
H2	−0.0716	0.1135	0.3979	0.033*	
N3	0.2723 (3)	0.1075 (2)	0.18987 (12)	0.0352 (3)	
N5	0.4521 (3)	0.4721 (2)	0.83639 (13)	0.0335 (3)	
N4	0.3980 (3)	0.1435 (2)	0.37169 (13)	0.0308 (3)	
C5	0.3058 (2)	0.18519 (19)	0.29330 (13)	0.0232 (3)	

### Atomic displacement parameters (Å^2^)

**Table:**

	*U*^11^	*U*^22^	*U*^33^	*U*^12^	*U*^13^	*U*^23^
Cl1	0.0377 (2)	0.0308 (2)	0.0310 (2)	0.01700 (17)	0.00766 (16)	0.00711 (15)
Cl2	0.0359 (2)	0.0417 (2)	0.0307 (2)	0.01407 (18)	0.00600 (16)	0.01193 (17)
O1G	0.0373 (7)	0.0351 (7)	0.0328 (7)	0.0125 (5)	0.0048 (5)	0.0066 (5)
C1G	0.0417 (12)	0.0504 (13)	0.0699 (16)	0.0099 (10)	0.0192 (11)	0.0091 (11)
S1	0.0392 (2)	0.0296 (2)	0.0249 (2)	0.02017 (17)	0.01270 (15)	0.01233 (15)
S2	0.0286 (2)	0.0409 (2)	0.02151 (19)	0.00569 (17)	0.00775 (15)	0.00506 (16)
C4	0.0239 (7)	0.0251 (7)	0.0206 (7)	0.0111 (6)	0.0049 (5)	0.0048 (6)
N1	0.0314 (7)	0.0223 (6)	0.0244 (6)	0.0086 (5)	0.0073 (5)	0.0064 (5)
N2	0.0342 (7)	0.0230 (6)	0.0238 (6)	0.0080 (5)	0.0072 (5)	0.0031 (5)
C3	0.0261 (7)	0.0244 (7)	0.0266 (8)	0.0038 (6)	0.0067 (6)	0.0050 (6)
C1	0.0227 (7)	0.0237 (7)	0.0213 (7)	0.0114 (6)	0.0048 (5)	0.0066 (5)
N6	0.0434 (9)	0.0308 (7)	0.0275 (7)	0.0142 (7)	0.0051 (7)	0.0009 (6)
C6	0.0317 (8)	0.0237 (7)	0.0216 (7)	0.0090 (6)	0.0048 (6)	0.0075 (6)
C2	0.0259 (7)	0.0240 (7)	0.0228 (7)	0.0042 (6)	0.0030 (6)	0.0003 (6)
N3	0.0454 (9)	0.0423 (8)	0.0237 (7)	0.0263 (7)	0.0087 (6)	0.0063 (6)
N5	0.0305 (8)	0.0345 (8)	0.0320 (7)	0.0130 (6)	0.0045 (6)	0.0044 (6)
N4	0.0393 (8)	0.0321 (7)	0.0255 (7)	0.0203 (6)	0.0062 (6)	0.0077 (6)
C5	0.0230 (7)	0.0230 (7)	0.0243 (7)	0.0081 (5)	0.0085 (5)	0.0074 (6)

### Geometric parameters (Å, °)

**Table:**

O1G—C1G	1.417 (3)	C1—C2	1.398 (2)
O1G—H1G	0.89 (3)	N6—C6	1.305 (2)
C1G—H1G1	0.9600	N6—H6B	0.90 (3)
C1G—H1G2	0.9600	N6—H6A	0.83 (3)
C1G—H1G3	0.9600	C6—N5	1.305 (2)
S1—C5	1.7603 (16)	C2—H2	0.9300
S1—C1	1.7775 (15)	N3—C5	1.306 (2)
S2—C6	1.7709 (17)	N3—H3A	0.92 (3)
S2—C4	1.7738 (15)	N3—H3B	0.89 (3)
C4—N2	1.329 (2)	N5—H5A	0.89 (2)
C4—C3	1.399 (2)	N5—H5B	0.90 (3)
N1—C1	1.326 (2)	N4—C5	1.315 (2)
N1—N2	1.3450 (19)	N4—H4B	0.91 (3)
C3—C2	1.366 (2)	N4—H4A	0.86 (3)
C3—H3	0.9300		
			
C1G—O1G—H1G	103.6 (19)	C6—N6—H6A	122.6 (19)
O1G—C1G—H1G1	109.5	H6B—N6—H6A	114 (2)
O1G—C1G—H1G2	109.5	N6—C6—N5	123.12 (17)
H1G1—C1G—H1G2	109.5	N6—C6—S2	115.38 (14)
O1G—C1G—H1G3	109.5	N5—C6—S2	121.38 (13)
H1G1—C1G—H1G3	109.5	C3—C2—C1	116.94 (14)
H1G2—C1G—H1G3	109.5	C3—C2—H2	121.5
C5—S1—C1	100.19 (7)	C1—C2—H2	121.5
C6—S2—C4	99.98 (8)	C5—N3—H3A	120.2 (16)
N2—C4—C3	123.90 (14)	C5—N3—H3B	120.0 (18)
N2—C4—S2	117.88 (11)	H3A—N3—H3B	120 (2)
C3—C4—S2	118.18 (12)	C6—N5—H5A	119.1 (15)
C1—N1—N2	118.93 (13)	C6—N5—H5B	117.8 (16)
C4—N2—N1	119.05 (13)	H5A—N5—H5B	123 (2)
C2—C3—C4	116.86 (15)	C5—N4—H4B	121.2 (17)
C2—C3—H3	121.6	C5—N4—H4A	122 (2)
C4—C3—H3	121.6	H4B—N4—H4A	116 (3)
N1—C1—C2	124.05 (14)	N3—C5—N4	123.08 (15)
N1—C1—S1	114.57 (11)	N3—C5—S1	115.75 (13)
C2—C1—S1	121.20 (12)	N4—C5—S1	121.17 (12)
C6—N6—H6B	123.2 (15)		
			
C6—S2—C4—N2	−37.51 (14)	C5—S1—C1—N1	126.26 (12)
C6—S2—C4—C3	144.58 (13)	C5—S1—C1—C2	−58.54 (14)
C3—C4—N2—N1	−5.3 (2)	C4—S2—C6—N6	134.58 (13)
S2—C4—N2—N1	176.96 (11)	C4—S2—C6—N5	−49.21 (15)
C1—N1—N2—C4	2.6 (2)	C4—C3—C2—C1	1.9 (2)
N2—C4—C3—C2	2.9 (2)	N1—C1—C2—C3	−4.5 (2)
S2—C4—C3—C2	−179.32 (13)	S1—C1—C2—C3	−179.24 (12)
N2—N1—C1—C2	2.3 (2)	C1—S1—C5—N3	149.37 (13)
N2—N1—C1—S1	177.35 (11)	C1—S1—C5—N4	−31.32 (15)

### Hydrogen-bond geometry (Å, °)

**Table:**

*D*—H···*A*	*D*—H	H···*A*	*D*···*A*	*D*—H···*A*
O1G—H1G···Cl1^i^	0.89 (3)	2.22 (3)	3.1038 (15)	171 (3)
N3—H3A···Cl2	0.92 (3)	2.28 (3)	3.1746 (17)	166 (2)
N3—H3B···O1G	0.89 (3)	1.95 (3)	2.839 (2)	171 (3)
N4—H4A···Cl1	0.86 (3)	2.70 (3)	3.3950 (16)	139 (2)
N4—H4B···Cl1^i^	0.91 (3)	2.36 (3)	3.2522 (15)	167 (2)
N5—H5A···Cl1	0.89 (2)	2.39 (2)	3.2614 (16)	170 (2)
N5—H5B···Cl2^ii^	0.90 (3)	2.25 (3)	3.1413 (17)	173 (2)
N6—H6A···Cl2^iii^	0.83 (3)	2.36 (3)	3.1878 (19)	175 (3)
N6—H6B···O1G^iv^	0.90 (3)	2.17 (2)	2.891 (2)	136 (2)

Symmetry codes: (i) −*x*+1, −*y*, −*z*+1; (ii) −*x*+1, −*y*+1, −*z*+1; (iii) −*x*, −*y*+1, −*z*+1; (iv) *x*, *y*+1, *z*+1.
